# Is virtual reality always an effective stressors for exposure treatments? some insights from a controlled trial

**DOI:** 10.1186/1471-244X-13-52

**Published:** 2013-02-11

**Authors:** Federica Pallavicini, Pietro Cipresso, Simona Raspelli, Alessandra Grassi, Silvia Serino, Cinzia Vigna, Stefano Triberti, Marco Villamira, Andrea Gaggioli, Giuseppe Riva

**Affiliations:** 1 Istituto Auxologico Italiano IRCCS, Applied Technology for Neuro-Psychology Laboratory, Milan, Italy; 2 Department of Psychology, Catholic University of Milan, Milan, Italy; 3 Iulm University, Milan, Italy

**Keywords:** Stress, Emotion elicitation, Virtual reality

## Abstract

**Background:**

Several research studies investigating the effectiveness of the different treatments have demonstrated that exposure-based therapies are more suitable and effective than others for the treatment of anxiety disorders. Traditionally, exposure may be achieved in two manners: in vivo, with direct contact to the stimulus, or by imagery, in the person’s imagination. However, despite its effectiveness, both types of exposure present some limitations that supported the use of Virtual Reality (VR). But is VR always an effective stressor? Are the technological breakdowns that may appear during such an experience a possible risk for its effectiveness?

**Methods:**

To answer these questions we compared changes following the exposure to an academic examination, one of the most universal examples of real-life stressors, in a sample of 39 undergraduate students. The same experience was offered using text (TX), audio (AU), video (VD), and VR. However, in the virtual environment we manipulated the experience introducing technological breakdowns. The Post Media Questionnaire (PMQ) and the Slater-Usoh-Steed Presence Questionnaire (SUS) were administered to each participant in order to evaluated self-report measures of anxiety and relaxation and the level of presence experience during media exposure. Electrocardiogram (ECG), Thoracic Respiration Signal (RSP) and Facial corrugator supercilii muscle Electromyography (EMG) were recorded in order to obtain objective measures of subjects’ emotional state.

**Results:**

Analyses conducted on PMQ showed a significant increase in anxiety scores and a mirror decrease in relax scores after all our emotional procedures, showing that all the condition were effective in inducing a negative emotional response. Psychometric scores and psychophysiological indexes showed that VR was less effective than other procedures in eliciting stress responses. Moreover, we did not observe significative difference in SUS scores: VR induced a sense of presence similar to that experienced during the exposition to other media.

**Conclusions:**

Technological breakdowns significantly reduce the possibility of VR eliciting emotions related to complex real-life stressors. Without a high sense of presence, the significant advantages offered by VR disappear and its emotional induction abilities are even lower than the ones provided by much cheaper media.

**Trial registration:**

Trial registration number: NCT01683617

## Background

Different research studies have shown that exposure-based therapies are more suitable and effective than others in the treatment of anxiety disorders [1-9].

Exposure is a process in which the patient is progressively exposed to the feared stimulus or the situation that provokes anxiety. Traditionally, exposure is experienced in two ways: by imagery in the person’s imagination, or in vivo, with direct contact to the stimulus.

Nevertheless, all roses have thorns, and this is true for both types of exposure, too. On one side, some patients report difficulties when asked to imagine the feared situation; furthermore, emotions have been shown to modulate visual imagery and perception [10], impairing visualization of detailed scenes. On the other side in vivo exposure is not fully under the control of the therapist and requires a high effort in terms of money and time expenditure.

To overcome these issues an emerging approach is the use of Virtual Reality Exposure Treatments (VRET) [11-15]. Compared to traditional methods, VR has the advantage to elicit a sense of presence in users, defined as the “feeling of being in a world that exists outside of the self” [16,17]. Another advantage of VR is that in such a setting, as in the real world, the subject can manipulate and interact with the environment differently from all the other techniques in which users are passive and not active [13,18]. It is also possible to grade the intensity of the stimulus following the personal needs of each patient. In this way, the patient feels less uncomfortable about the treatment and his/her motivation increases [19]. Recently different quantitative meta-analyses, have been conducted on studies reporting VRET treatments. Parson and Rizzo [20] analyzed data from 21 studies who have evaluated anxiety and/or phobia before and after VRET. They conclude that VRET has a statistically large effect on all affective domains, and thus it is a relevant approach to reduce anxiety-related symptoms. They conclude that VRET has a statistically large effect on all affective domains, and thus it is a relevant approach to reduce anxiety-related symptoms. Similarly, Powers and Emmelkamp [21] provide effect size estimates for virtual reality treatment in comparison to in vivo exposure and other control conditions. They found a predictable larger effect of VRET compared to the control conditions; but more interestingly, VRET outperformed in vivo exposure.

But is VR always an effective stressor? Are the technological breakdowns that may appear during such an experience a possible risk for its effectiveness?

Unfortunately only a few studies compared the efficacy of VR in inducing an emotional response with other media and/or with real-life reactions [22-24]. These studies also have the limitation to investigate very simple emotive reactions, not so complicated as might be a response related to a real-life stressor. Recently, Hartanto and colleagues investigated the effects of three different interruption mechanisms on subjective experience during a VRET session for social phobia with scripted avatar-patient dialogues [25]. Specifically, the study examined three interruption mechanisms: (1) dialogue dependent, that occurs freely in the flow of the dialogue; (2) speech dependent, that occurs during the pauses in the dialogue; and (3) context independent, that is randomly. Although, the dialogue dependent interruption seems superiors on the perceived dialogue flow, on the user preference and on the dialogue replies, participants rated presence highest for the context independent mechanism. According to Riva and Mantovani [26] indeed, we are present in an environment - real and/or virtual - when we are able, inside it, to intuitively transform our intentions in actions.

Based on the current status of presence research, much uncertainty remains about its usefulness for emotional responses induction and VRET. Only weak evidence for a relationship between presence and emotional responses has been found [22,27], and no study has yet addressed this relationship. In other words, it is still unclear whether VR with low measured presence is characterized by a reduced effectiveness in inducing negative emotional responses compared to other traditional procedures.

According to these theoretical premises, we manipulated the level of presence by creating a virtual environment with two technical breakdowns: the head tracking was randomly reversed for 20 seconds during the experience and the avatars’ lips didn't move at the same time as their voice. We argued that these two kinds of technical breakdowns reduce the capability of VR in supporting the intentions of the user and its capability to elicit emotive reactions.

Hence, we compared changes following the exposure to an academic examination, one of the most universal examples of real-life stressors, in a sample of 39 undergraduate students. The same experience was offered using text, audio, video, and “impoverished” VR.

## Methods

### Aim of the study

The general goal of this study was to compare the efficacy of different media in eliciting negative emotional responses. In particular, we wanted to examine if technological breakdowns that appeared during VR could reduce its effectiveness in comparison with other media (text, audio, and video) in arousing emotions related to a real-life stressor.

To compare the efficacy of these techniques, we compared the relative effectiveness of different presentation modalities of the same stressor (an oral academic examination).

In particular the following hypotheses were tested:

a. The four experimental situational emotional induction procedures are effective in eliciting a negative emotional response in undergraduate students.

b. The four experimental situational emotional induction procedures elicit different intensities of emotional response.

c. The VR experience, when associated to technology breakdowns, is less effective than other media in eliciting the negative emotional response.

d. “Impoverished” VR induces a sense of presence similar to those experienced during the exposure to other media.

### Participants

The experimental sample included 41 students. The mean age of the sample was 21.15 ± 3.6, with a mean years of school education of 13.59 ± .498.

Students were recruited from the University of IULM, Italy. In order to be included in the study, students had to meet the following criteria: (1) absence of heart, blood pressure, or major medical diseases; (2) Age between 20 and 30 years; (3) Absence of pharmacotherapy or other medications that might interfere with the measures being assessed (e.g, psychoactive medications; antihypertensives); (4) Absence of psychiatric disorders; (5) Absence of neurological diseases, mental retardation, psychosis, alcohol or drug dependence; (6) No migraine, headache, or vestibular abnormalities. Subject eligibility was determined via subject-reported medical history.

Of these, two did not fulfill the inclusion criteria and were excluded. Therefore, 39 students were included in the statistical analysis of psychometric questionnaires, while only 34 were included in the physiological one.

Before participating in the study, each participant was provided with written information about the study and was invited to give written consent for the inclusion. Participants were also asked to not smoke or assume caffeine during the day of the experiment. The study received ethical approval by the Ethical Committee of the Istituto Auxologico Italiano.

### Negative emotions elicitation

According to an emotional narrative validated in a preliminary study [28], we created an audio, a text, a video and a virtual environment based on that script. The emotional imagery narrative was based on methods developed by Lang and his colleagues [29] and further adapted by Sinha and colleagues [30,31]. In the emotional imagery script, alluding to an oral academic examination, specific details of the situation were elicited referring to specific stimulus and response particulars, including physical and interpersonal context details, verbal and cognitive attributions regarding the people involved. A sample of the script used in this study follows above:

“It’s your last exam before graduating. The next exam session will be only the next year. You know that, if you fail, you will have to wait some months to graduate. You feel very nervous. In the exam’s room you see four of your classmates. They are talking about the examination and they seem to be very worried and troubled. They tell you that all of them failed the exam and that the professor is very strict. Your anxiety increases. You feel your breath becoming heavy. Your heart is faster and faster (…)”.

### Laboratory assessment

#### Psychometric assessment

The following psychometric questionnaires were administered to each participant at the start of the experiment and after each experimental condition:

•*Post Media Questionnaire* (PMQ, [32,33])*.* The Post Media Questionnaire (PMQ) was used to assess the emotions elicited by media exposure. In the questionnaire participants rate the greatest emotions experienced during the media experience (anxiety, disgust, anger, fury, surprise, relax, happiness, sadness) on a 7-point likert scale

•*Slater-Usoh-Steed Presence Questionnaire* (SUS, [34]): The Presence Questionnaire was used to assess the level of presence experience during media exposures. In the questionnaire participants rate the characteristics of the media experience (feeling of being there, realism, involvement) and on a 7-point likert scale.

#### Psychophysiological assessment

At the beginning, during and at the end of the laboratory session, electrocardiogram (ECG), Thoracic Respiration Signal (RSP) and Facial corrugator supercilii muscle Electromyography (EMG) were recorded in order to obtain objective measures of subjects’ emotional state.

Collected data were analyzed using Matlab 7.0 (The Mathworks, Natick, MA). Cardiovascular and respiratory activities were monitored to evaluate both voluntary and autonomic effect of respiration on heart rate, analyzing both inter-beat (R-R) interval extracted from electrocardiogram and respiration (from a chest strip sensor). The respiration signal was filtered to produce a smooth sinusoidal signal. The Respiration Rate (RSP_Rate) index represents the peak-to-peak time (max-to-max distance of the sinusoid). Furthermore, following the guidelines of Task force of the European Society of Cardiology and the North American Society of Pacing and Electrophysiology, a typical Heart Rate Variability (HRV) spectral method indexes were used to evaluate the autonomic nervous system response [35-40]. At this purpose spectral analysis was performed using Fourier spectral methods with custom software. The rhythms have been classified as very low frequency (VLF, <0.04 Hz), low frequency (LF, from 0.04 to 0.15 Hz), and high frequency (HF, from 0.15 to 0.5 Hz) oscillations. This procedure enabled us to calculate the HF index [39,40].

The raw electromyography is a collection of positive and negative electrical signals; their frequency and amplitude give us information on the contraction or rest state of the muscle. Amplitude is measured in μV (micro-Volts). As the subject contracts the muscle, the number and amplitude of the lines increases; as the muscle relaxes, it decreases. We considered the EMG Root Mean Square (EMG_RMS) for rectifying the raw signal and converting it to an amplitude envelope. In this study, we were not interested in frequency related to muscle fatigue.

To summarize psychophysiological measures used in this study:

•HF: High Frequency, 0.15 to 0.5 Hz oscillations in R-R interval extracted from Electrocardiogram. In stressful situations this index tends to decrease from baseline.

•EMG_RMS: Root Mean Square EMG from facial corrugator supercilii muscle Electromyography. In stressful situations this index tends to increase from baseline

•RSP_Rate: peak-to-peak time from Respiration signal. In stressful situations this index tends to increase from baseline

Psychophysiological data were obtained using the NeXus-4 equipment, with the BioTrace+ software for recording signals, developed by Mind Media. The module gets analog data coming from different physiological sensors and, after conditioning and digitalizing, sends them to a host Personal Computer (PC) using a wireless connection.

### Experimental protocol

A within-subjects design was used to compare self-reported anxiety and relax measures from a pre-condition baseline to each of the four experimental conditions. Participants acted as their own controls.

Specifically, the study compared the following conditions:

•*Condition 1: Virtual Reality (VR).* In the VR condition participants were asked to wear a head-mounted display (Vuzix VR Bundle with twin high-resolution 640x480 LCD displays, 920,000 pixels, iWear® 3D compliant) in order to have a 3D view of the virtual scene. The virtual environment was rendered using a portable computer (ACER ASPIRE with CPU Intel® Core™i5 and graphic processor Nvidia GeForce GT 540M). Participants also had a joystick (Xbox Controller), which allowed them to explore and to interact with the environment. The virtual scene represented an academic oral examination in a classroom (the virtual environment is included in NeuroVR 2.0 [23], a free open source software that was previously used in different emotion inducing experiments [22,24]. In all of them the scenes of the environment were able to elicit a strong emotional response and feeling of presence that was similar [22] or even higher [24] than the real scene. Based on these previous studies, we assumed that the virtual scene without technical breakdowns would be very effective in eliciting negative emotional response. In the selected scene the individual was asked to enter in the class and meet a group of students who commented on the difficulty of the exam and how the professor was very tough. Then, he/she had to approach the professor who was just rejecting a student. Finally his/her name was called by the professor and asked to start the exam. During the experience the participant also experienced two technological breakdowns: the head tracking was randomly reversed for 20 sec during the experience and the students’ lips didn't move at the same time as their voice. The complete experience was about 3 minutes long.

•*Condition 2: Audio* (AU). Subjects were asked to imagine themselves as vivid as possible during an academic oral examination, following an audio narrative matching the VR experience. Script development procedures was based on methods developed by Lang and his colleagues [29], and further adapted and validated in our previous study [28]. During the imagery exposure subjects were asked to close their eyes and to imagine the situation described. The audio narrative was about 3 minutes long and was played through the computer speakers. (Compliant Standards High Definition Audio)

•*Condition 3: Texts* (TX). Based on the script used in the Imagery Exposure, a script in a written form was developed. Participants were asked to read a text in which an academic oral examination is described. The script was defined with sufficient details to induce subjects to enter in the story, as hypothesized by Geerig [41]. The text was read in about 3 minutes on the 17” computer screen.

**Figure 1:**
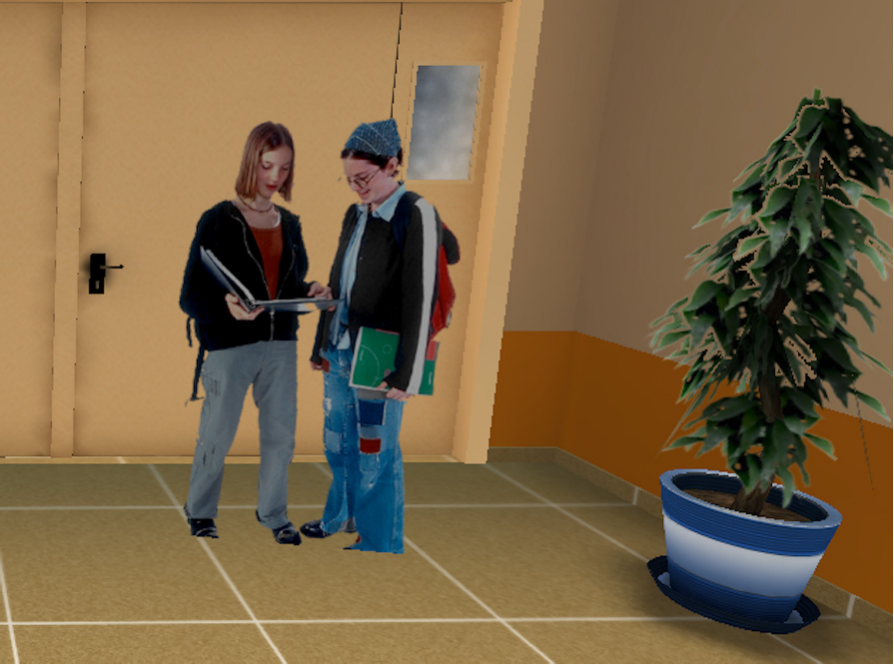
**Screenshot from VR condition.** Figure illustrates virtual environment representing an academic oral examination in a classroom.

•*Condition 4: Video (VD).* In the VD condition participants were exposed to a video, based on the Imagery Exposure script, and using the same actors involved in the VR scene, showing the same situation described before. The video was about 3 minutes long, too. Participants watched the video on the 17" (1920x1200) computer screen (Figure 1).

Participants were randomly assigned to the order in which conditions were presented. The order of presentation of each experimental condition was counterbalanced for each participant following an established randomization schema obtained from http://www.randomizer.org/.

Before starting the experiment each participant was provided with written information about the study and was invited to give written consent for the inclusion. Then, each participant was submitted to a subject-reported medical history interview.

**Figure 2:**
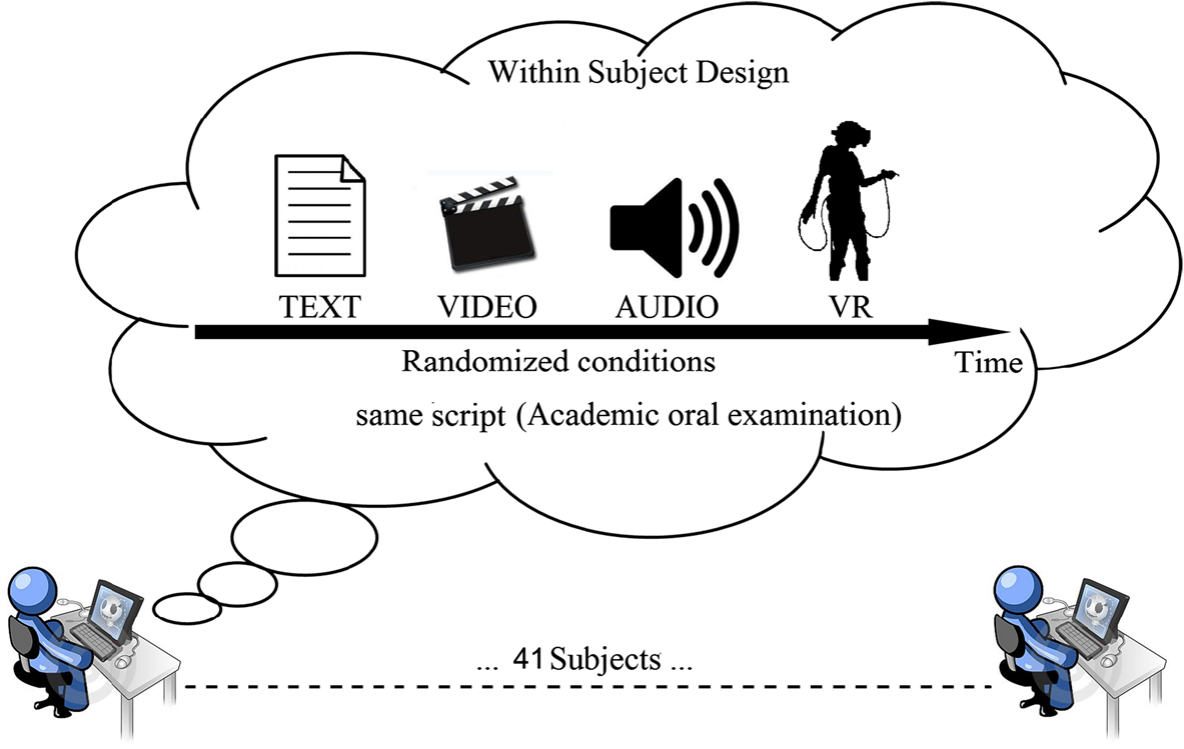
Time scheduled of the experiment.

At the start of the experimental session, participants were seated at a desk in front of a computer monitor. The eye-to-display distance was about 0.75 meters. Participants were connected with biosensors for recording their psychophysiological parameters (heart rate, respiration, and facial corrugator supercilii muscle activity). A baseline measure of these signals was registered for 3 minutes in rest condition, with eyes opened. Once the physiological baseline was recorded, the experimental session started and psychophysiological signals were recorded until the end of all the tasks. Then, in order to measure the psychological variations occurring during the different exposure conditions, subjects completed an adapted version of the PMQ in order to assess their perceived level of anxiety and relax immediately before and after each condition. After each exposure condition they completed also the SUS to assess presence’s level experienced. Before each condition there was a 3 min baseline during which participants were asked to stay completely relaxed with open eyes, while their physiological parameters were recorded (Figure 2).

## Results

Data were entered into Microsoft Excel and analyzed using SPSS and STATA. Change in psychometric and physiological measures within subjects were calculated using repeated measure ANOVAs.

### Psychometric variables

To confirm that the stress induction was successful and that participants were stressed and experienced a negative emotional state after the exposure to each condition (VR, AU, VD, TX), we evaluated self-report measures of anxiety and relaxation assessed through PMQ before and after each condition. A 2 (time) × 4 (condition) repeated measures ANOVAs on the mean scores were conducted in order to compare the effect of time on anxiety and relax scores depending on the specific condition.

Regarding anxiety, results showed a significant main effect of time, F (1,36) = 44.2, p < .001, ηp2 = .522. In particular, the mean score showed before (M = 2.51, SD = .168) was lower than that assessed after (M = 4.29, SD = .275) the exposure to conditions. The main effect of condition was non-significant, F (3,108) = 2.56, p = ns, ηp2 = .066. However, the interaction effect between time x condition was significant, F (3,108) = 4.93, p < .05, ηp2 = .121, suggesting that anxiety mean scores changed over time depending on condition (Table 1).

**Table 1:** Mean and Standard deviation of anxiety scores assessed trough the PMQ Questionnaire before and after all the four conditions

	** Time**	
**Condition**	**Pre**	**Post**
**Virtual Reality**	2.65 (1.16)	3.78 (1.88)
**Video**	2.41 (1.18)	4.27 (1.83)
**Audio**	2.51 (1.3)	4.76 (1.94)
**Text**	2.49 (1.23)	4.35 (1.94)

A mirror result was obtained regarding relax level. Results showed a significant main effect of time, F (1,36) = 41.1, p < .001, ηp2 = .533. In particular, the mean scores showed before (M = 4.16, SD = .162) was higher than that assessed after (M = 2.82, SD =. 233) the exposure to conditions. The main effect of condition was non-significant, F (3,108) = .887, p = ns, ηp2 = .024. However, the interaction effect between time x condition was significant, F (3,108) = 2.75, p < .01, ηp2 = .07, indicating that relax mean scores changed over time depending on condition (Table 2).

**Table 2:** Mean and Standard deviation of relax scores assessed trough the PMQ Questionnaire before and after all the four conditions

	** Time**	
**Condition**	**Pre**	**Post**
**Virtual Reality**	4.16 (1.14)	2.84 (1.6)
**Video**	4.14 (1.25)	3 (1.87)
**Audio**	4.24 (1.34)	2.43 (1.53)
**Text**	4.14 (1.22)	3.03 (1.6)

To test for simple effects, we calculated the mean difference of self-report anxiety measures (score at the baseline minus score after condition) for each condition. A repeated measure ANOVA with condition as the within factor revealed a significant main effect of condition in anxiety scores, F (3,114) = 4.59, p < .005, and in relax scores, F (3,114) = 2.82, p < .05 (Table 3).

**Table 3:** Mean and Standard Deviation of main difference in anxiety and relax scores (score at the baseline minus score after condition)

	**Condition**						
	**Virtual Reality**	**Audio**	**Video**	**Text**	***F***	***η***_***p***_^***2***^	***p***
**Anxiety**	-1.02 (.325)	-2.28 (.322)	-1.64 (.37)	-1.82 (.32)	4.59	.525	.000***
**Relax**	1.07 (2.03)	1.89 (1.41)	1.12 (1.52)	1.28 (1.94)	2.82	.069	.042*

***p < 0.001, **p < 0.01, *p < 0.05.

In particular, repeated-measures t-tests (using a Bonferroni adjustment α = .05/3 = .017) showed that the mean difference in anxiety scores was significantly lower in the VR condition (M = -1.02, SD = .325) than in the AU condition (M = -2.28, SD = .322), t(36) = -1.256, p < .01, ES = .385;

Then, in order to evaluate self-report measures of presence, we analyzed the SUS total score (sum of item 1, 2 and 3) assessed after each condition. A one-way ANOVA on the mean scores was conducted in order to test whether SUS scores changed depending on the specific condition.

Analyses showed no significant main effect of condition, F(3,111) = .084, p = ns (Table 4).

**Table 4:** SUS total score after each condition

**Condition**	**Virtual Reality**	**Audio**	**Video**	**Text**	***F***	***p***
**Presence**	12.1 (4.03)	12.4 (4.15)	12.3 (4.35)	12.1 (3.75)	.084	.969

ANOVA did not reveal a significative main effect of condition.

### Psychophysiological variables

A repeated measures analysis of variances was also conducted in order to test whether psychophysiological indexes changed depending on the specific experimental procedures.

Due to complexity of signal processing some subjects turned in a problematic bio signal, composed by many artifacts and/or missing values. Thus, some subjects have been excluded from analysis, in particular we excluded nine subjects form HF computation and eight subjects from EMG_RMS and RSP_Rate computation. So, eventually, the psychophysiological statistical analyses have been performed on 32 or 33 subjects, a number well-recognized to be of good level in these kinds of studies. More, a statistical power analysis confirmed the fairness of our sample size (Table 5).

**Table 5:** Differences in main effects for HF, EMG_RMS and RSP_Rate

**Measure**	**(I) Condition**	**(J) Condition**	***Mean difference (I-J)***	***Std. error***	***p***
**HF**	Virtual Reality	Audio	5.554	1.916	.041*
		Video	5.477	1.719	.020*
		Text	3.745	2.338	.714
**EMG_RMS**	Virtual Reality	Audio	-5.489	1.092	.001***
		Video	-3.61	1.133	.020*
		Text	-4.898	1.032	.001***
**RSP_Rate**	Virtual Reality	Audio	-3.626	.604	.001***
		Video	-2.22	.460	.001***
		Text	-2.808	.585	.001***

This table reports the comparison between Virtual Reality and the other stimuli (Audio, Video, Text), showing statistical significant differences for all the three measures. ***p < 0.001, **p < 0.01, *p < 0.05.

**Figure 3:**
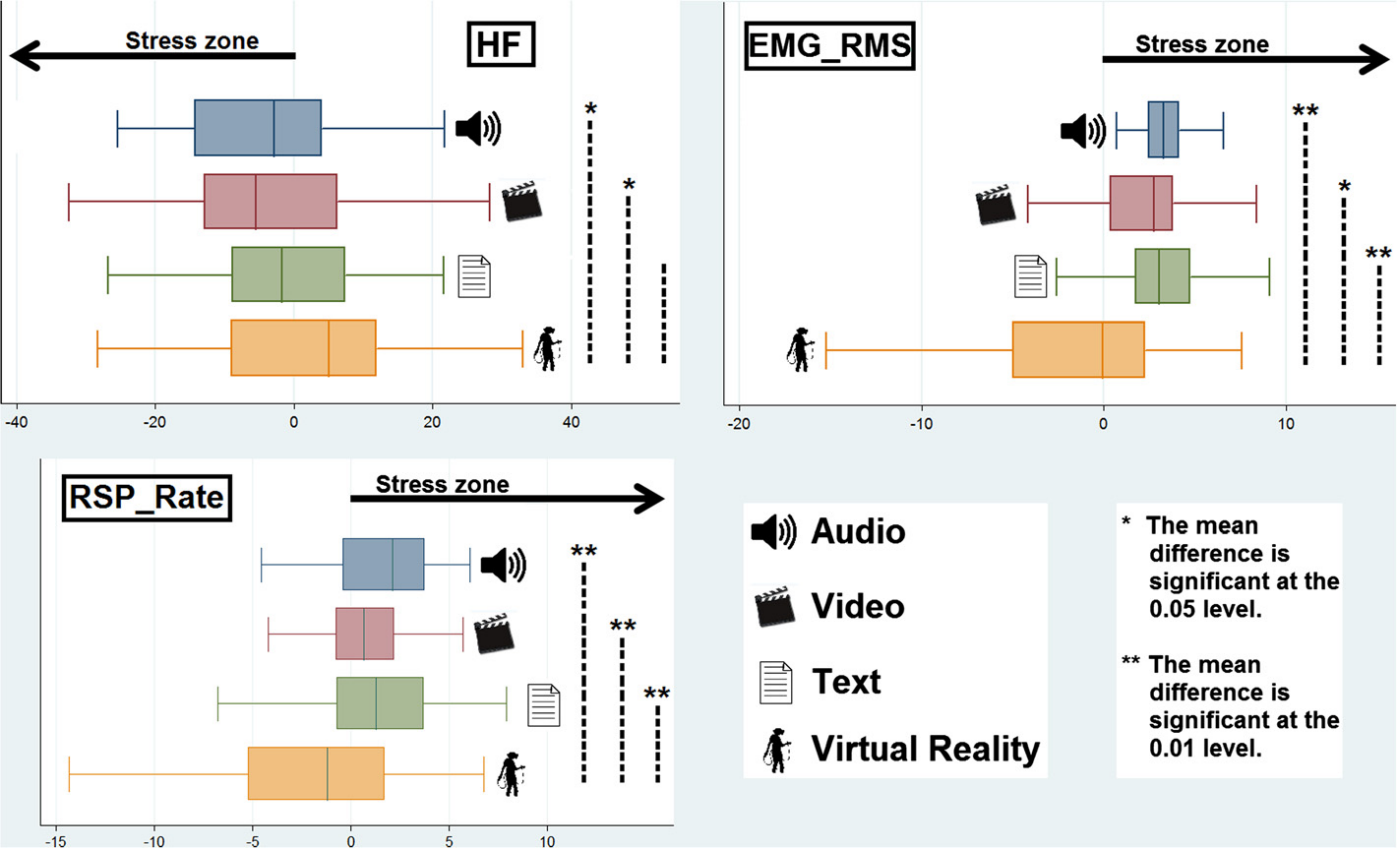
**A comparison within conditions with significance level showed accordingly.** HF, EMG_RMS and RSP. Rate graphics per each condition, showing deviation from baseline. The arrow (Stress zone) indicates the sense of the variation to indicate an increasing in stress in that direction. It is clear from Figure 3 that Virtual Reality condition differs from other conditions in the direction quality, confirming that technological breakdowns significantly reduce the possibility of Virtual Reality of eliciting emotions related to complex real-life stressors, besides the stressful script used.

Results showed a main effect of condition for HF, F(96,3) = 3.16, p < .028, EMG_RMS, F(99,3) = 16.1, p < .001, and RSP_Rate, F(99,3) = 17.1, p < .001. In particular, adjustment for multiple comparisons, with Bonferroni correction, showed the significant differences reported in Table 4 (Figure 3).

## Discussion

Virtual Reality Exposure Treatment (VRET) is an emerging approach for the treatment of anxiety disorders that offers different advantages over classical imaginative and in vivo exposure. Recently, different quantitative meta-analyses have been conducted on studies reporting VRET treatments supporting their efficacy in reducing anxiety-related symptoms [11,12].

But is VR always an effective stressor? Are the technological breakdowns that may appear during such an experience a possible risk for its effectiveness? Different studies suggested a possible link between emotional response and the level of presence experienced in a virtual environment. For example, Michaud and colleagues [42] experimentally manipulated presence in a sample of heights phobics who had to take an elevator and perform tasks on a scaffold outside of a 15-story building. When the immersion in the virtual environment was conducted in a high-presence setting, the level of anxiety was significantly higher than when the immersion was conducted in a low-presence setting.

Riva and colleagues [43] explored, too, the relationship between presence and emotions experienced in VR. Their data showed a circular interaction between presence and emotions: on one side, the feeling of presence was greater in the "emotional" environments; on the other side, the emotional state was influenced by the level of presence.

Taken together these results underline the existence of a bi-directional relationship between presence and emotions. However, no previous studies investigated if the reduction of presence induced by technological breakdowns may be accompanied by a reduced results efficacy of VR as a stressor for exposure treatments.

To answer this question we compared changes following the exposure to an academic examination in a sample of 39 undergraduate students. The same experience was offered using text, audio, video, and VR. However, in the virtual environment we manipulated the experience introducing two technological breakdowns: the head tracking was randomly reversed for 20 seconds during the experience and the avatars’ lips didn't move at the same time.

In the study we first investigated if our emotional induction procedures were really effective in eliciting an emotional stress-related response. Results, in accordance with our hypothesis, showed that conditions were effective in inducing a stress-related response. We found a significant increase in anxiety scores measured through PMQ and a mirror decrease in relax scores after all our emotional procedures.

In addition, we assumed that the four emotive induction procedures elicits different intensity of responses and, in particular, we hypothesized that VR, when experienced with breakdowns, was less effective than traditional procedures. As hypothesized, results showed significative differences in the psychometric scores and psychophysiological correlates between the four experimental conditions. In particular, results on anxiety scores assessed through the PMQ showed that negative emotional response elicited through VR was less effective than AU procedure. Psychophysiological indexes confirmed this interesting result and, moreover, showed that VR was less effective in inducing a stress-related response compared to the other conditions. Results, in fact, showed an increased level from baseline in EMG_RMS index, a well-known index of emotional valence, in AU, VD and TX conditions, clearly indicate a higher negative emotion for the participants. On the other hand, VR condition showed no increased level from baseline. Also RSP_Rate showed similar results, indicating an accelerated respiration pattern in AU, VD and TX condition and a decelerated one in the VR condition. This confirms that Virtual Reality containing technological breakdowns has not been able to induce stress, besides the negative script. Finally, a further confirmation arises from HF, an important index of Heart Rate Variability. Decreases in HF is a well-recognized index of stress, more it has been recognized as an index that works well with different kind of stress, from a more cognitive working load to a situational stress due to psychological individual perception of environmental demands that exceed one's own adaptive ability to meet them [44]. In our study, lower level of HF for Audio, Video, and Text indicate a stress measured by the means of this cardiovascular index. Virtual Reality, on the opposite, showed an increased level. Thus also for this index we can confirm that technological breakdowns in Virtual Reality worked well.

Finally, results confirmed our fourth hypothesis. As we expected, “impoverished” VR induced a sense of presence similar to that experienced during the exposition to other media. This data confirms the hypothesis that technological breakdowns impacted the VR efficacy in inducing a sense of presence in the user, creating break in presence [26,44]. Moreover, this fact was associated in our study with less effectiveness of VR in eliciting a negative emotional response compared to other media.

## Conclusions

In conclusion, even if using VR it is possible to experience more presence than in real life [24,45], or to generate a body transfer illusion [46], it is also possible to experience less presence than reading a text or watching a video. Therefore, if the focus is on designing applications capable of eliciting emotions with the goal of reducing or modifying them (as in psychological therapy), the environments must be able to induce a high feeling of presence. For this reason an important goal of VR developer is to guarantee that the virtual experience offered to their user is smooth and transparent enough to provide a high level of presence through a full support to the intentions of the user. Without it, the significant advantages offered by VR disappear and its emotional induction abilities are even lower than the ones provided by much cheaper media.

## Abbreviations

AU: Audio; ECG: Electrocardiogram; EMG: Electromyography; EMG_RMS: Electromyography Root Mean Square; HF: High Frequency; HRV: Heart Rate Variability; LF: Low Frequency; μV: Micro-Volts; PC: Personal Computer; PMQ: Post Media Questionnaire; R-R: Inter-beat; RSP: Thoracic Respiration Signal; RSP_Rate: Respiration Rate; SUS: Slater-Usoh-Steed Presence Questionnaire; TX: Texts; VD: Video; VLF: Very Low Frequency; VR: Virtual Reality; VRET: Virtual Reality Exposure Treatments

## Competing interests

The authors declare that they have no competing interests.

## Authors’ contributions

FP prepared the first draft of the manuscript. SR, AG and FP supervised the study in its clinical aspect. FP, GR, SS and ST collected literature material and supervised the background of the study. PC supervised the psychophysiological aspect of the study. CV created the study technological materials. GR, PC and SS developed the first draft manuscript into the final version suitable for publication. MV, AG and GR conceived the idea of the study and supervised its scientific design. All authors read and approved the final manuscript.

## Pre-publication history

The pre-publication history for this paper can be accessed here:

http://www.biomedcentral.com/1471-244X/13/52/prepub
